# Acute respiratory effects and biomarkers of inflammation due to welding-derived nanoparticle aggregates

**DOI:** 10.1007/s00420-017-1209-z

**Published:** 2017-03-03

**Authors:** Katrin Dierschke, Christina Isaxon, Ulla B. K. Andersson, Eva Assarsson, Anna Axmon, Leo Stockfelt, Anders Gudmundsson, Bo A. G. Jönsson, Monica Kåredal, Jakob Löndahl, Joakim Pagels, Aneta Wierzbicka, Mats Bohgard, Jörn Nielsen

**Affiliations:** 1 Division of Occupational and Environmental Medicine, Department of Laboratory Medicine, Lund University, 221 85 Lund, Sweden; 2 Ergonomics and Aerosol Technology, Department of Design Sciences, Lund University, Lund, Sweden; 3 Occupational and Environmental Medicine, Gothenburg University, Gothenburg, Sweden

**Keywords:** Occupational exposure, Exhaled breath condensate, Nasal lavage, Leukotriene B4, Interleukin 6 and 8

## Abstract

**Purpose:**

Welders are exposed to airborne particles from the welding environment and often develop symptoms work-related from the airways. A large fraction of the particles from welding are in the nano-size range. In this study we investigate if the welders’ airways are affected by exposure to particles derived from gas metal arc welding in mild steel in levels corresponding to a normal welding day.

**Method:**

In an exposure chamber, 11 welders with and 10 welders without work-related symptoms from the lower airways and 11 non-welders without symptoms, were exposed to welding fumes (1 mg/m^3^) and to filtered air, respectively, in a double-blind manner. Symptoms from eyes and upper and lower airways and lung function were registered. Blood and nasal lavage (NL) were sampled before, immediately after and the morning after exposure for analysis of markers of oxidative stress. Exhaled breath condensate (EBC) for analysis of leukotriene B4 (LT-B4) was sampled before, during and immediately after exposure.

**Results:**

No adverse effects of welding exposure were found regarding symptoms and lung function. However, EBC LT-B4 decreased significantly in all participants after welding exposure compared to filtered air. NL IL-6 increased immediately after exposure in the two non-symptomatic groups and blood neutrophils tended to increase in the symptomatic welder group. The morning after, neutrophils and serum IL-8 had decreased in all three groups after welding exposure. Remarkably, the symptomatic welder group had a tenfold higher level of EBC LT-B4 compared to the two groups without symptoms.

**Conclusion:**

Despite no clinical adverse effects at welding, changes in inflammatory markers may indicate subclinical effects even at exposure below the present Swedish threshold limit (8 h TWA respirable dust).

## Introduction

Welders are a large group of workers exposed to gases and particles which can cause adverse effects in airways such as bronchitis, asthma and metal fume fever (Antonini et al. 2003). Furthermore, the immune system may be affected, with impaired resolution of local inflammation and reduced capacity to eliminate infectious agents (Zeidler-Erdely et al. 2012). Welding in mild steel accounts for the majority of all welding (Taube 2013). Although such welding has often been looked upon as less harmful than other forms of welding, an increasing number of studies show adverse effects also at mild steel welding even at moderate exposure levels (Lillienberg et al. 2008; Jönsson et al. 2011, 2015; Hedmer et al. 2014).

Exposure at mild steel welding is complex and varies depending on welding method, electrode used and the material being welded. The particles generated are often in the nano-size range, initially between 10 and 100 nm while later aggregating to larger complexes (Zimmer and Biswas 2001).

There is growing evidence that particles in the nano range (i.e. <100 nm) may be more toxic than bigger ones, at equal mass concentration, because of their high surface-to-mass ratio and smaller radius which make them more chemically reactive and also more potent inducers of oxidative stress and subsequent inflammation (Ferin et al. 1992; Oberdörster et al. 2005; Tran et al. 2000; Donaldson et al. 2002; Renwick et al. 2004).

However, welders may also be exposed to larger particles, e.g. at grinding the welded material (Zimmer and Maynard 2002). In an earlier study, we found that although the symptoms in welders increased significantly during working days the association with the welding exposure was not clear (Jönsson et al. 2015). As the welders also ground the welded material, it was difficult to conclude to what degree the symptoms were elicited by the welding fumes. Such knowledge is important for the preventive work.

The objective of this study was to clarify if exposure to fumes from gas metal arc welding in mild steel in a level found in the industry in Sweden today affects the airways of welders with or without work-related lower airway symptoms.

## Materials and methods

### Study population

A panel of welders was selected from six welding companies in southern Sweden. The panel consisted of a group of welders with work-related symptoms from the low airways in the last month (WS) and another group of welders without such symptoms (WNS). A third group of non-welding subjects without low airways symptoms (NWNS) was also included. Nobody was current smokers (never smokers or ex-smokers for at least 5 years) and they had no regular medication.

All the welders in the companies were asked to fill in a self-administered screening questionnaire (Drexler et al. 1999) containing questions regarding employment, exposure, smoking habits, allergies and work-related symptoms from eyes and airways. Work-related symptoms were defined as symptoms where recovery occurred during weekends and holidays (Ferris 1978). Thirty-four (25%) out of 134 welders had experienced work-related symptoms from the lower airways (wheezing, dyspnoea or cough) the last month. Of this group, 21 subjects were eligible for the WS group. They were consecutively asked to participate. Participants to the WNS group were matched to the symptomatic ones as good as possible with regard to company, total welding time, age, earlier smoking and atopy. The participants to the NWNS group were chosen from workers in a storeroom in one of the welding factories or from another mechanical industry without welding exposure. These subjects were not exposed to respiratory irritants. As the number of female welders was small only male subjects were selected.

The age, the total time as a welder and results from the basal medical examination (as described below) are given in Tables 1 and 2. Nobody has diagnosed airway diseases. Subjects with acute symptoms from the eyes and airways such as common cold were not allowed to participate. The study was carried out outside the pollen season.

**Table 1:** Age, lifetime welding, smoking habits, skin-prick test (SPT) positivity and lung function, forced vital capacity (FVC) and forced expiratory volume in the first second (FEV_1_), in percent of expected and work-related symptoms from eyes and nose at least once a week the last month in welders with symptoms from the lower airways (WS), welders with no symptoms (WNS) and in non-welders with no symptoms (NWNS) at the basal examination before the study

	WS *N* = 11	WNS *N* = 10	NWNS *N* = 11
Age, years (median; min, max)	42 (29–66)	51 (34–66)	47.5 (29–66)
Welding time, years (median; min, max)	14 (2–39)	31 (2–43)	–
Smoking habits (*N*; %)			
Present	0	0	0
Never	5 (45.5)	4 (40)	8 (72.7)
SPT positivity (*N*; %)	3 (27.3)	1 (10.0)	1 (9.1)
Lungfunction (median; min, max)			
FVC %	99.2 (72–114)	94.0 (80–117)	99.8 (89–121)
FEV_1_ %	101.0 (66–132)	93.4 (75–128)	104.1 (91–129)
Symptoms (*N*)			
Eyes	1	1	1
Nose	6^a^	1	2

^a^ *p* = 0.05 between WS and NWNS

**Table 2:** The welders with symptoms from the lower airways during the last month, skin-prick test positivity for a standard panel of aero allergens, metacholine test (MCH) positivity and lung function (FVC and FEV_1_ as % of predicted) at the basal medical examination before the study

	Nasal	Lower airways	Skin-prick test	MCH PD_15_^a^	FVC%	FEV_1_%
1	No	Dyspnoea	Negative	192	114	114
2	Yes	Dyspnoea, cough	Positive (birch, timothy, mug wort)	nc	93	101
3	No	Cough	Negative	nc	101	100
4	Yes	Cough	Negative	nc	72	74
5	Yes	Wheezing breath	Negative	49	79	71
6	Yes	Dyspnoea, cough, wheezing	Negative	nc	105	108
7	No	Cough	Positive (house mites)	nc	99	101
8	Yes	Dyspnoea, wheezing	Positive (timothy, cat, dog)	3	82	66
9	Yes	Cough	Negative	nc	85	90
10	No	Dyspnoea, cough, wheezing	Negative	nc	114	132
11	No	Dyspnoea, cough	Negative	–^b^	108	127

*nc* Not calculable

^a^MCH PD_15_ is defined as the provocative dose that cause a 15% decrease in the FEV_1_ from the baseline. A positive test is defined as PD_15_ < 167 µg

^b^Test inhibited by cough

The study was approved by the regional ethical committee at Lund University (H4 160/2007) and performed according to the Declaration of Helsinki. An informed written consent was obtained from all participants prior to exposure.

### Study design

The participants were randomly exposed twice at different occasions either to welding fume or to filtered air (FA) respectively, in a double-blind manner in an exposure chamber. The average time between the two exposures were two weeks (min–max: 1–8 weeks). Each exposure time was 5.5 h with a lunch break for 1 h after 2.5 h of exposure. Exposure occurred on Mondays, ensuring that the welders had not been exposed to welding fume for at least 48 h prior to the exposure. The participants went through medical examinations including lung auscultation, spirometry and acoustic rhinometry before and immediately (30–60 min) after exposure. Symptoms and peak expiratory flow (PEF) were registered before, during (10 min, 1 h 10 min, 2 h 45 min, 4 h 10 min, 5 h 10 min after start) and immediately after the exposure. To study markers of oxidative stress nasal lavage (NL), urine- and blood samples were obtained before and immediately after the exposure and, if practically possible, also the morning after (*N* = 30 persons after welding exposure, *N* = 28 persons after FA exposure). Furthermore, exhaled breath condensate (EBC) was sampled before the exposure, at the beginning of the lunch break, and immediately after the exposure. The subjects were in rest during the exposures.

### Exposure chamber and exposure

The chamber conditions and generation system of welding fumes and FA are described in detail in Isaxon et al. (2013). In short, the chamber was a stainless steel room (21.6 m^3^) having a capacity of comfortably keeping three participants sitting at the same time. It was supplied with clean air through a separate conditioning system by which air flow, temperature and relative humidity could be controlled.

Particles for exposure were derived from metal active gas (MAG) welding in mild steel using a shielding gas mixture Ar/CO_2_ (82/18%) and a 1 mm in diameter electrode with a feeding rate of 3 cm/s. The welding was carried out in a box located in an adjacent room out of sight for the test subjects and the medical staff. Welding was performed for 3 min in a 20-min cycle throughout the exposure event. This non-uniform exposure resembles real work situations. The welding fume was supplied to the clean air flow just prior to entering the chamber. The time from generation of welding fume to the chamber was 2 s. Continuous monitoring and averaging of the mass concentration with a custom-built computer program made it possible to increase or decrease the welding time of each pulse, as well as the pulse frequency, toward the desired mean concentration. During the exposures, PM_2.5_ particle mass concentration in the chamber was monitored with a Tapered Element Oscillating Microbalance (TEOM) with a cyclone as a precollector for particles larger than 2.5 µm. The particle number concentration and mobility size distribution (10–650 nm) were measured using a Scanning Mobility Particle Sizer (SMPS) system. The chemical composition of the welding aerosol was characterized by PIXE and X-ray energy dispersive spectroscopy (XEDS).

Generation of welding fume followed the same time schedule, whether the participants were exposed to welding fume or to FA only. In the case of FA exposure, the generated welding fume was bypassed the exposure chamber.

The procedure proved to deliver reproducible results throughout the number of exposure events, with a mean particle number concentration of 64,300/cm^3^ and a standard deviation between the mean values of different exposure events (SDoM), of 15,000, PM_2.5_ mass concentration 1000 µg/m^3^ (SDoM: 70), CO_2_ 670 ppm (SDoM: 134), RH 26.9% (SDoM: 1.7) and temperature 23.3 °C (SDoM: 1.0). The geometric count median diameter of particles was 160 nm with a variation between the different events up to ±10%. The count mean diameter of the number size distributions is 190 nm, with the 5th and 95th percentiles of 47 and 570 nm, respectively.

The median mass concentration during all exposure events was 946 µg/m^3^, with the 1st and 3rd quartiles 633 and 1307 µg/m^3^, respectively. The elemental composition in mass % was iron 70%, and manganese 20%. The remaining 10% contained silica (4.5%) and metals such as copper and zinc (Isaxon et al. 2013).

### Basal medical examination

Before the exposure the participants went through a general medical examination including standardized questions about past and present respiratory and cardio-vascular diseases and allergies (Tables 1, 2). They were also asked for specific symptoms from eyes and airways including dry and productive cough. Chronic bronchitis was defined according to the Medical Research Council Committee (1965). A standard skin-prick test and a spirometry were also performed (see below). Participants with symptoms from the lower airways went through a methacholine test (MCH) for the assessment of the nonspecific bronchial hyper-reactivity (Table 2). This test could not be carried out in one participant because of cough. Nobody turned out to have any current disease including chronic bronchitis and allergic symptoms. Because of asthma-like symptoms one subject was prescribed salbutamol when necessary.

### Medical methods

Symptoms were recorded using a visual analogue scale (Molen 1995; Dorman et al. 2007). Symptoms from the eyes (scratching, burning or itching), the nose (running, itching/sneezing, blocked and bleeding), the pharynx (sensation of dryness, irritation and/or itching, hoarseness/rough voice and mucus), and from lower airways (attacks of dyspnoea, chest tightness, wheezing breath, and dry or productive cough) were registered.

Skin-prick test (SPT) was carried out on the volar part of the forearm using 12 aeroallergens [birch, grasses (Phleum prat., Dactylis glom., Arrhenatherum elat.), mugwort, cat, dog, horse, house dust mite (Dermatophagoides pteronyssinus), moulds (Cladosporium herb., Alternaria alt., Aspergillus fum.)], and read after 15 min (ALK Abello A/S, Copenhagen, Denmark). Reaction sizes were recorded in relation to histamine (10 mg/ml), according to Aas and Belin (1973).

PEF was measured with a Mini Wright Flow Meter (measuring range of 60–800 L/min), three recordings each time. The best one will be taken for the statistical analysis (Brusasco et al. 2005; Miller et al. 2005).

Spirometry was performed with SPIRARE 3 (DIAGNOSTICA, Oslo, Norway) according to the European Respiratory Society, ERS (Miller et al. 2005; Quanjer et al. 1993). Vital capacity (VC) and Forced expiratory flow in the first second (FEV_1_) were obtained and compared to a standard material (Berglund et al. 1963).

MCH was performed according to Larsson et al. (2007). In short, a jet nebulizer (Spira Elektro 2; Respiratory Care Center, Hämeenlinna; Finland) was used. Methacholine chloride in saline was administered in six successive cumulative doses (2, 10, 40, 160, 460 and 1360 μg). FEV_1_ was measured at 1 and 3 min after each inhalation step. The person was considered hyper-reactive when FEV_1_ fell more than 15% compared to the baseline at a total dose of 167 μg or less (Larsson et al. 2007).

Acoustic rhinometry was performed using a RhinoScan v. 2.5 (Interacoustics A/S, Assens, Denmark) according to the producers advises with the participant sitting with fixed neck. The minimum cross-sectional area between 0 and 22 mm from nares and between 22 and 54 mm (MCA 1 and MCA 2, respectively) and the nasal volume between these distances (Volume 1 and Volume 2, respectively) were recorded (Hilberg 2002). The mean value of three consecutive measurements for each nostril was registered and the result is given as the sum.

EBC was sampled using an RTube (Respiratory Research, Charlottesville, VA, USA). The subject took 15 deep breathes into the RTube which was cooled to –25 °C. The condensate was then allocated to tubes in amounts of 50 µl each and frozen at −80 °C for later analysis.

NL was carried out as earlier described (Xu et al. 2013) using 18 ml 37 °C 0.9% NaCl solution. After cell separation the pellet was suspended in a volume of 500 μl by a 0.9% NaCl solution, and 200 μl each were transformed to glass slides using a cytospin centrifuge (600 rpm for 5 min, Cytospin 2, Shandon Inc. Pittsburgh, PA, USA).The slides were air dried. The supernatant was pipetted to two Cryovial tubes prepared with inhibitor according to Cho et al. (2003) and frozen at −80 °C. It was not possible to get NL from one of the participants.

### Laboratory analyses

The NL cells were stained by the May-Grünewald-Giemsa method. A total of 100 granulocytes were counted and the share of eosinophils was recorded. The reading was blindly performed.

Blood samples were analyzed for hemoglobin and a differential count of white blood cells was performed using Sysmex XE-5000 and Sysmex XE-1800i. Fibrinogen was analyzed in plasma by using Sysmex CA-7000 according to the instruction of the manufacturer. C-reactive protein (CRP) was analyzed in plasma by using the instrument Cobas c501. The analyses were performed at the Division of Clinical Chemistry at Lund University Hospital.

The cytokines ICAM, IL-6, IL-8 and TNF-α in nasal lavage, and IL-6 and IL-8 in serum were analysed using a multiplexed immunoassay Luminex-platform (Bio-Plex 200, Bio-Rad Life Science, Hercules, CA). Commercially available kits were purchased from Bio-Rad (Bio-Rad Life Science, Hercules, CA). The analysis was performed according to the instructions of the manufacturer. Bovine serum albumin (BSA) was added to nasal lavage samples and standards to a final concentration of 0.5% BSA. All samples were analysed in duplicates. Samples with values below the limit of detection (LOD) were awarded in the statistical analysis a value equal to half LODs. The LODs were in nasal lavage 0.43 for IL-6, 0.42 for IL-8, 1.5 for TNF-α and 7.8 for ICAM; in serum 0.44 for IL-6 and 0.35 for IL-8 (pg/ml).

The concentration of 8-oxo-21-deoxyguanosine (8-oxo-dG) in urine was measured using liquid chromatography tandem mass spectrometry with prior purification using solid-phase extraction according to Teichert et al. (2009) with some modifications (Engström et al. 2010). Detection limit was 10 pg/ml and the coefficient of variation in a quality control sample at 9 ng/ml was 10%.

Leukotriene B4 in exhaled air condensate was analysed using commercial LT-B4 EIA kit from EnzoLife Sciences (Enzo Life Science AG, Lausen, Switzerland) according to the protocols supplied by the manufacturer. The analyses were performed at the Division of Occupational and Environmental medicine, Lund University.

Club cell protein (CC16) in serum was analysed at the Department of Occupational and Environmental medicine in Gothenburg, using commercial ELISA kits from BioVendor (BioVendor Laboratory Medicine, Inc., Brno, Czech Republic), according to the protocols supplied by the manufacturer.

### Statistics

Differences between welding and FA exposure for single time points were assessed by odds ratios (ORs) with 95% confidence intervals (CIs), estimated by logistic regression. Changes over time were investigated using repeated measures mixed models (continuous outcomes) or general linear models with repeated measures (categorical outcomes).

Possible interactions between exposure (welding fume/filtered air) and subgroups (WS, WNS and NWNS) were assessed by adding an interaction term between the exposure and the subgroup.

When the outcome variables are ln-transformed, it is not entirely clear how the results from the statistical analyses should be interpreted. Therefore, the point estimates and associated confidence limits have been transformed with the formula 100 × (e^B^−1). The results can then be interpreted as the percentage increase/decrease in mean in the evaluated group (Vittinghoff et al. 2005).

For continuous outcomes where it was necessary to use non-parametric tests, differences between welding and filtered air exposure were analysed with Friedman’s two-way analysis of variance. For such analyses, it was not possible to adjust for the order of exposure or examine interaction effects. For those variables that cannot be analysed with general linear models we present only group medians. All analyses were performed in SPSS 18.0.

## Results

### Symptoms

Symptoms were frequently reported both before welding and FA exposure. A significant decrease in nasal symptoms was registered at welding exposure at 4 h 10 min and onward (Table 3). No other consistent changes during the exposures were found regarding number of persons with symptoms or in intensity of symptoms as studied from the VAS scales (data not shown). No significant interactions between exposure and subgroups were noticed.

**Table 3:** Number of persons with nasal symptoms at different time points at welding and filtered air (FA) exposure and odds ratios (OR) between welding and FA and the 95% confidence interval (CI, upper and lower bound)

	Welding *N* (%)	FA *N* (%)	OR (95% CI)	*p* value
Before exposure	18 (56)	15 (47)	1.46 (0.70, 3.05)	0.31
After start				
10 min	13 (41)	13 (41)	1.00 (0.59, 1.70)	1.00
1h 10 min	12 (38)	11 (34)	1.16 (0.62, 2.17)	0.65
2h 45 min	10 (31)	13 (41)	0.66 (0.42, 1.04)	0.07
4h 10 min	8 (25)	14 (44)	0.43 (0.23, 0.80)	0.01
5h 10 min	10 (31)	15 (47)	0.51 (0.30, 0.89)	0.02
5h 30 min	9 (28)	14 (44)	0.50 (0.25, 0.99)	0.05

### Medical examinations

No significant changes from before to immediately after exposure were found regarding lung auscultation in the whole group and in the subgroups at welding or filtered air exposure (data not shown).

In rhinometry no significant changes were noticed in the total group at welding compared to FA exposure (Table 4). Significant interactions between exposure and subgroups were found for all studied parameters (MCA 2 *p* = 0.04; Volume 1 *p* = 0.02 and Volume 2 *p* = 0.03) except for MCA 1 which was close to significant (*p* = 0.06; results for Volume 1 and 2 are shown in Table 4). The WS group increased most in all measures indicating a nasal opening during welding exposure whereas the NWNS group tended to decrease.

**Table 4:** Rhinometry before and after, and the changes (Δ) (mean, standard deviation) from before to immediately after exposure to welding fumes and filtered air (FA), respectively, in the total group and in the subgroups of welders with symptoms from the lower airways (WS), welders with no symptoms (WNS) and in healthy non-welders (NWNS)

	Volume 1 (cm^3^)				Volume 2 (cm^3^)			
	Total group	WS	WNS	NWNS	Total group	WS	WNS	NWNS
Welding								
Before	4.37 (0.74)	4.50 (0.53)	4.44 (0.97)	4.18 (0.72)	8.27 (3.23)	7.02 (1.78)	9.40 (4.80)	8.48 (2.32)
After	4.49 (0.77)	4.78 (0.60)	4.49 (0.90)	4.20 (0.74)	9.81 (4.17)	9.56 (2.89)	10.95 (4.49)	9.03 (5.03)
Δ before to after	0.12 (0.23)	0.28 (0.19)	0.05 (0.16)	0.01 (0.24)	1.55 (2.78)	2.54 (2.20)	1.55 (2.52)	0.55 (3.36)
FA								
Before	4.38 (0.75)	4.49 (0.67)	4.49 (0.87)	4.16 (0.73)	8.57 (3.59)	8.01 (2.50)	9.79 (3.33)	8.01 (4.64)
After	4.51 (0.69)	4.60 (0.57)	4.53 (0.91)	4.40 (0.62)	9.00 (3.42)	7.80 (2.40)	10.67 (3.80)	8.68 (3.60)
Δ before to after	0.13 (0.32)	0.11 (0.26)	0.04 (0.34)	0.24 (0.35)	0.43 (1.96)	-0.21 (2.45)	0.89 (1.84)	0.67 (1.47)

A positive result means an increase in the parameter studied from before to after exposure

*p*-values for interaction between the exposure and the subgroups: *p* = 0.02 (Volume 1), *p* = 0.03 (Volume 2)

No effects of welding exposure were registered by spirometry or by PEF in the whole group and in the subgroups (data not shown).

### Blood

**Fig. 1:**
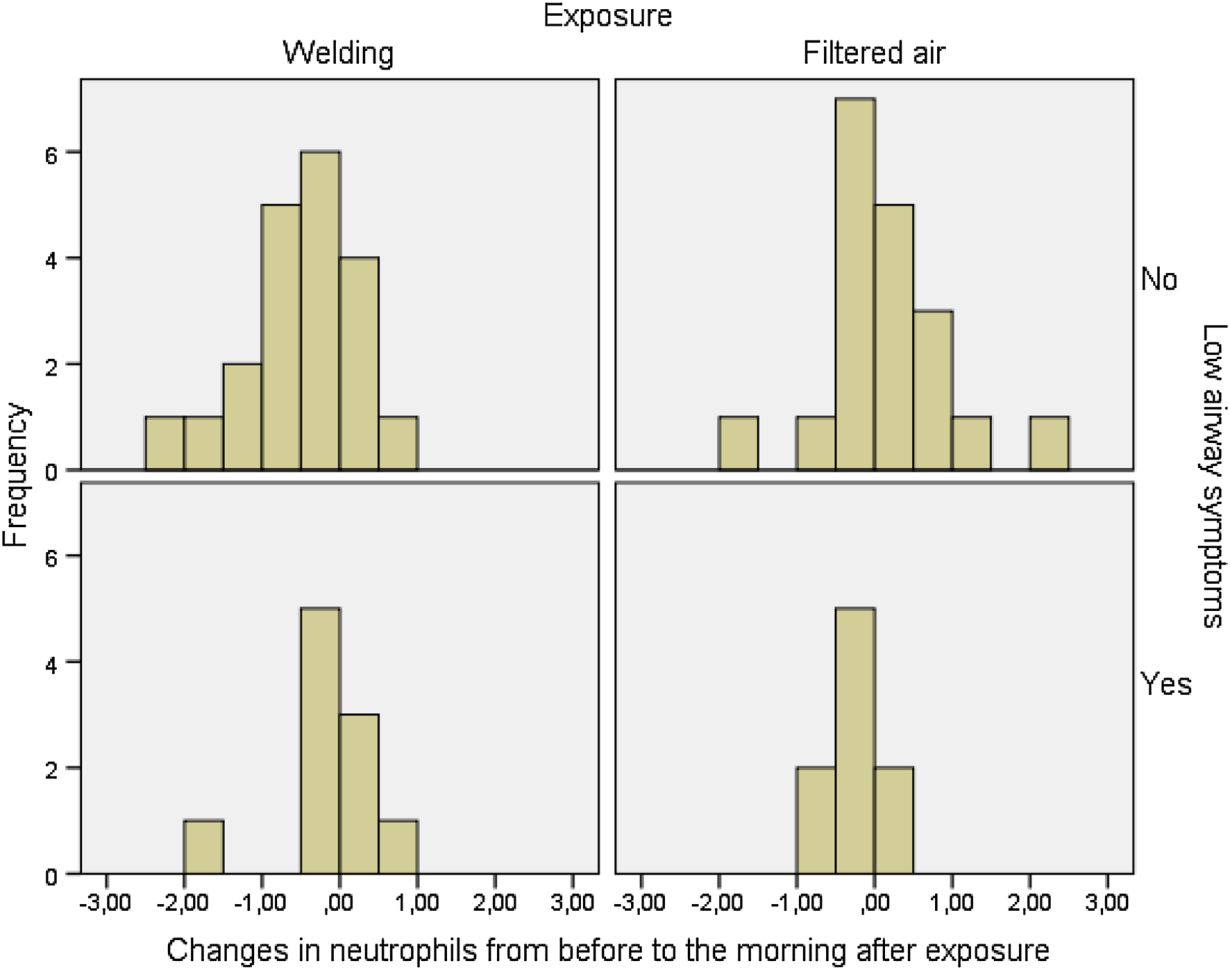
The changes in neutrophils in blood (10^9^/l) from before to the morning after exposure to welding fume and filtered air respectively in subjects with and without low airway symptoms. A positive value means an increase from before to after exposure

In the total group, neutrophils did not change immediately after welding exposure compared to FA (Table 5). A tendency to interact between the exposure and the levels of neutrophils in the subgroups was noticed with an increase predominantly in the WS group immediately after welding exposure (Table 5). On the contrary, the day after exposure a significant decrease was noticed in the whole group, most clearly in the two non-symptomatic groups (Fig. 1). No significant changes were associated with welding exposure compared to FA regarding lymphocytes, eosinophils and monocytes in the total group. Neither were effects of bronchial reactivity, atopy or life time welding noticed (data not shown).

**Table 5:** The levels in blood neutrophils before, immediately after and the morning after the exposure to welding and filtered air (FA), and the changes (Δ) (mean, standard deviation), respectively, in the total group and in the subgroups of welders with symptoms from the lower airways (WS), welders with no symptoms (WNS) and in healthy non-welders (NWNS)

	Blood neutrophils	Total group	WS	WNS	NWNS
Welding	Before	3.59 (1.11)	3.38 (0.50)	4.11 (1.76)	3.32 (0.65)
	After	3.71 (0.81)	3.96 (0.89)	3.53 (0.92)	3.62 (0.62)
	Morning after	3.22 (0.79)	3.20 (0.62)	3.41 (1.14)	3.08 (0.60)
	ΔBefore to after	0.12 (0.95)	0.57 (0.88)	−0.58 (0.98)	0.30 (0.63)
	ΔBefore to morning after	−0.43 (0.69)	−0.22 (0.61)	−0.89 (0.77)	−0.24 (0.55)
FA	Before	3.47 (1.36)	3.51 (0.63)	3.74 (1.96)	3.18 (1.31)
	After	3.63 (1.50)	3.67 (0.70)	3.75 (1.77)	3.47 (1.91)
	Morning after	3.48 (1.47)	3.08 (0.44)	3.65 (1.67)	3.67 (1.88)
	ΔBefore to after	0.16 (0.77)	0.16 (0.72)	0.01 (0.56)	0.29 (0.99)
	ΔBefore to morning after	−0.04 (0.75)	−0.36 (0.42)	−0.43 (0.59)	0.49 (0.79)

*p* values for interaction between the exposure and the subgroups: *p* = 0.08 (before to after) and *p* = 0.09 (before to the morning after)

**Fig. 2:**
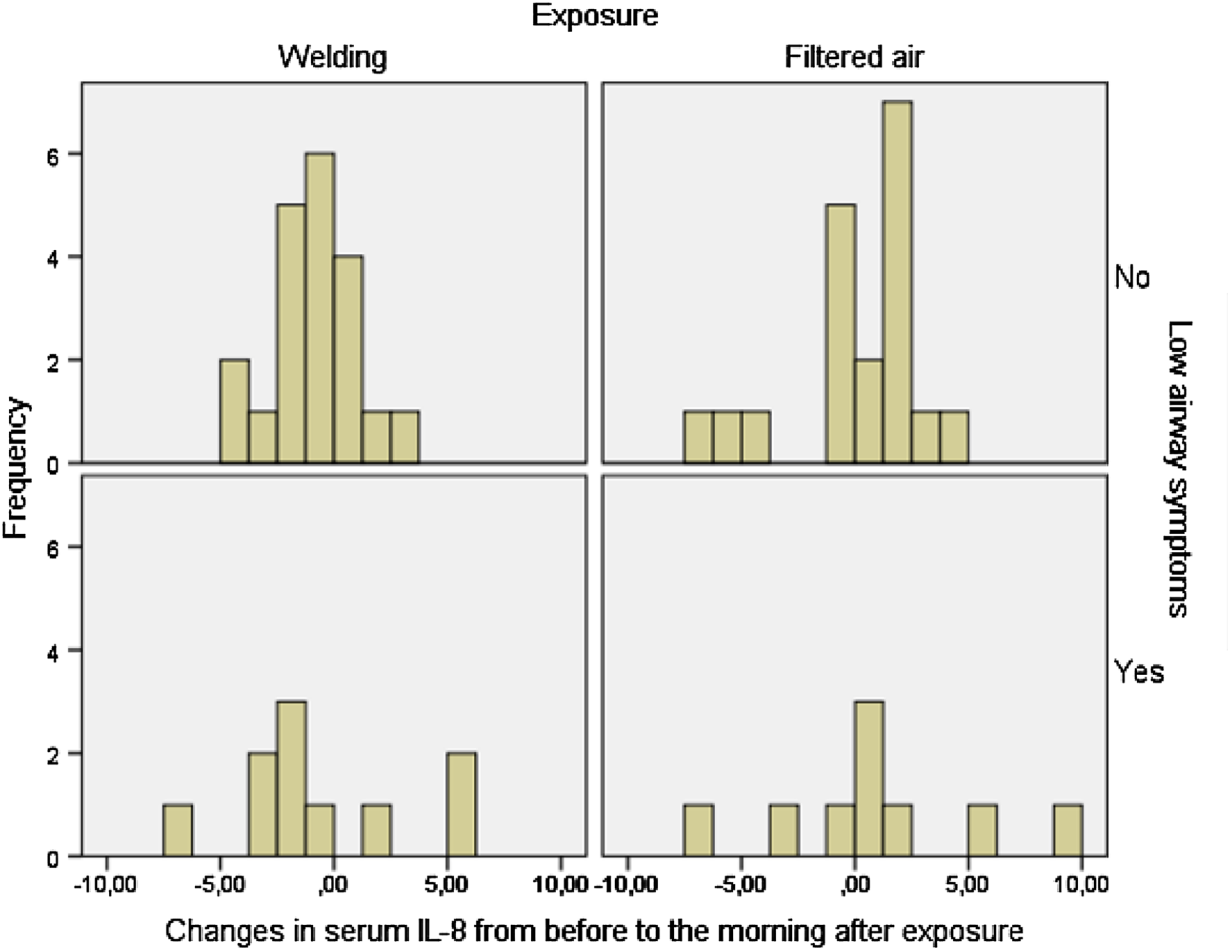
The changes in serum IL-8 (pg/ml) from before to the morning after exposure to welding fume and filtered air respectively in subjects with and without low airway symptoms. A positive value means an increase from before to after exposure

Serum IL-8 decreased significantly in the morning after welding in the total group compared to FA exposure (Table 6). The decrease was mainly seen in the WS group (Fig. 2). There were no significant changes in fibrinogen, C-reactive protein, CC16 and IL-6 in the total group and in the subgroups. Neither were effects of bronchial reactivity, atopy or life time welding noticed (data not shown).

**Table 6:** The levels in IL-8 in serum (pg/ml) before, immediately after and the morning after the exposure to welding and filtered air (FA), and the changes (Δ) (mean, standard deviation), respectively, in the total group and in the subgroups of welders with symptoms from the lower airways (WS), welders with no symptoms (WNS) and in healthy non-welders (NWNS)

	IL-8 in serum	Total group	WS	WNS	NWNS
Welding	Before	6.34 (0.0, 21.9)	7.09 (0.0, 20.3)	6.50 (3.0, 12.6)	5.28 (2.7, 21.9)
	After	6.35 (3.4, 24.1)	6.39 (4.6, 24.1)	7.13 (3.4, 13.8)	5.36 (4.2, 18.4)
	Morning after	5.23 (0.0, 26.1)	5.60 (0.0, 26.1)	5.42 (2.8, 11.1)	4.82 (0.0, 21.1)
	ΔBefore to after	0.23 (−4.57, 4.85)	0.00 (−2.28, 4.44)	0.57 (−4.57, 4.85)	0.33 (−3.52, 1.76)
	ΔBefore to morning after	−1.06 (−7.33, 5.87)	−1.59 (−7.33, 5.87)	−1.00 (−3.09, 3.08)	−0.81 (−3.86, 2.16)
FA	Before	6.31 (0.0, 21.6)	5.50 (0.0, 21.6)	6.48 (3.3, 11.9)	6.31 (2.5, 19.8)
	After	6.68 (0.0, 26.1)	6.44 (0.0, 16.9)	6.32 (3.7, 13.1)	8.18 (3.3, 26.1)
	Morning after	6.82 (0.0, 16.6)	6.98 (4.4, 10.5)	7.22 (5.6, 16.6)	6.57 (0.0, 14.3)
	ΔBefore to after	0.31 (−5.95, 6.35)	0.00 (−5.95, 3.23)	0.13 (−1.88, 1.98)	1.69 (−3.66, 6.35)
	ΔBefore to morning after	0.20 (−6.87, 9.57)	0.00 (−6.79, 9.57)	1.54 (−1.07, 4.68)	−0.53 (−6.87, 2.30)

A positive result means an increase in the parameter studied from before to after the exposure

*p* values for interaction between welding and FA: *p* = ns (before to after) and *p* = 0.01 (before to the morning after)

*ns* not significant

### NL, EBC and urine

**Fig. 3:**
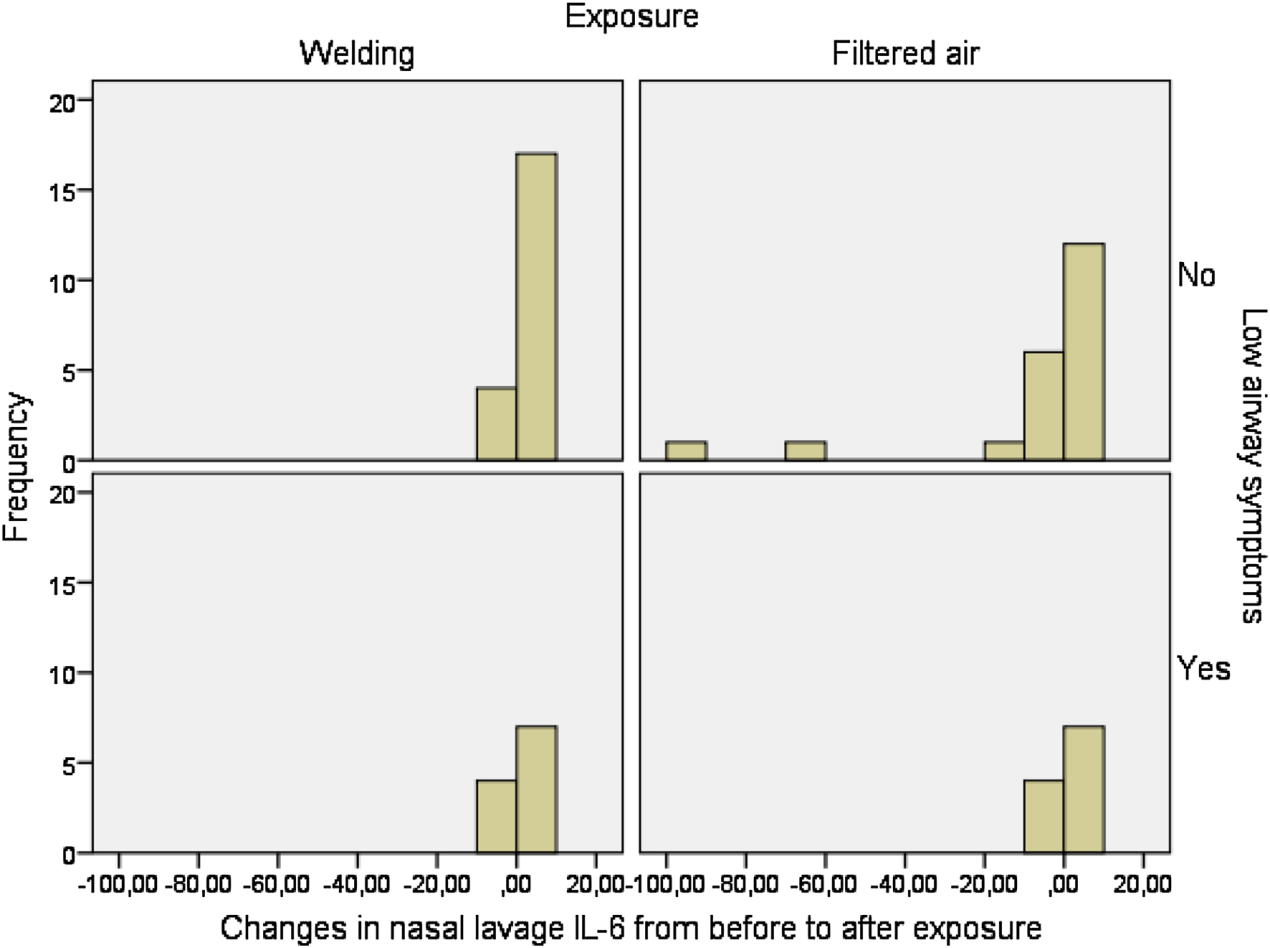
The changes in nasal lavage IL-6 (pg/ml) from before to immediately after exposure to welding fume and filtered air respectively in subjects with and without low airway symptoms. A positive value means an increase from before to after exposure

There was a significant increase in NL IL-6 immediately after welding compared to FA exposure in the total group (*p* = 0.02), but this increase was only seen in the asymptomatic subgroups (Table 7; Fig. 3). No effects of welding were seen in the morning after. No effects of welding fumes were seen in IL-8 or in the numbers of eosinophilic cells, ICAM and TNF-α (data not shown).

**Table 7:** The levels in IL-6 in nasal lavage (pg/ml) before, immediately after and the morning after the exposure to welding and filtered air (FA), and the changes (Δ) (mean, standard deviation), respectively, in the total group and in the subgroups of welders with symptoms from the lower airways (WS), welders with no symptoms (WNS) and in healthy non-welders (NWNS)

	IL-6 in nasal lavage	Total group	WS	WNS	NWNS
Welding	Before	0.80 (0.0, 45.5)	1.04 (0.0, 4.6)	0.32 (0.0, 45.5)	1.01 (0.0, 8.6)
	After	1.22 (0.0, 47,0)	0.67 (0.0, 6.0)	1.46 (0.0, 47.0)	1.73 (0.0, 10.4)
	Morning after	0.92 (0.0, 31.4)	0.84 (0.0, 8.3)	1.20 (0.0, 31.4)	0.88 (0.0, 4.7)
	ΔBefore to after	0.28 (−1.72, 8.05)	0.00 (−1.34, 1.43)	0.90 (−0.42, 4.20)	0.54 (−1.72, 8.05)
	ΔBefore to morning after	0.00 (−14.10, 8.14)	0.00 (−0.84, 3.66)	0.31 (−14.10, 8.14)	−0.20 (−4.80, 0.80)
FA	Before	1.17 (0.0, 152.2)	1.33 (0.0, 4.8)	1.05 (0.0, 25.7)	1.54 (0.0, 152.2)
	After	1.28 (0.0, 60.3)	0.98 (0.0, 3.0)	1.33 (0.0, 11.7)	4.09 (0.0, 60.3)
	Morning after	1.53 (0.0, 88.9)	1.17 (0.0, 3.2)	0.88 (0.0, 85.1)	4.04 (0.0, 88.9)
	ΔBefore to after	0.00 (−91.88, 3.61)	0.00 (−3.50, 0.36)	0.11 (−14.02, 1.17)	0.00 (−91.88, 3.61)
	ΔBefore to morning after	0.06 (−83.34, 59.37)	0.13 (−2.88, 1.85)	0.27 (−0.55, 59.37)	0.00 (−83.34, 3.83)

A positive result means an increase in the parameter studied from before to after the exposure

*p* values for interaction between welding and FA: *p* = 0.02 (before to after) and *p* = ns (before to the morning after)

*ns* not significant

Regarding EBC, the WS group had about 10 time’s higher LT-B4 value than the other two asymptomatic subgroups before exposure to welding fume as well as to filtered air (Table 8). LT-B4 decreased during welding, but not at FA exposure in the whole group. Immediately after exposure the difference between welding and FA was significant (*p* = 0.02). The decrease was noticed in all the subgroups.

**Table 8:** LT-B4 (pg/ml, median, min, max) in exhaled breath condensate before, at half time and immediately after exposure to welding and filtered air (FA), respectively, in the total group and in the subgroups of welders with symptoms from the lower airways (WS), welders without symptoms (WNS) and in healthy non-welders (NWNS)

	LT-B4 in EBC	Total group	WS	WNS	NWNS
Welding	Before	50.0 (6.0, 504.0)	228.0 (6.0, 504.0)	29.0 (7.0, 447.0)	27.0 (7.0, 337.0)
	At half time	38.5 (6.0, 473.0)	309.0 (8.0, 473.0)	32.0 (11.0, 238.0)	20.0 (6.0, 238.0)
	After	28.0 (2.0, 431.0)	171.0 (12.0, 431.0)	17.5 (7.0, 328.0)	10.0 (2.0, 52.0)
FA	Before	70.0 (2.0, 632.0)	204.0 (8.0, 560.0)	31.0 (6.0, 437.0)	20.0 (2.0, 632.0)
	At half time	43.0 (0.0, 792.0)	263.0 (0.0, 510.0)	24.5 (12.0, 792.0)	24.0 (4.0, 738.0)
	After	79.5 (5.0, 758.0)	328.0 (6.0, 729.0)	79.0 (6.0, 296.0)	17.0 (5.0, 758.0)

*p* values for the differences between welding and FA: *p* = 0.48 (before), *p* = 0.08 (at half time) and *p* = 0.02 (after)

We saw no effects of welding exposure on 8-oxo-dG in urine, whether it was creatinine- or density adjusted, neither in the whole group nor in the subgroups after and the morning after the exposure (data not shown).

## Discussion

In this study of acute effects of mild steel welding fume exposure in welders with and without work-related symptoms from lower airways and in non-welders, we found no consistent increase in symptoms. Contrary, welders, especially those in the WS group, had a tendency to improve in nasal symptoms and in rhinometry during the exposure. Despite the lack of clinical effects, changes in some biomarkers at welding exposure compared to FA (blood neutrophils and IL-8 in serum, IL-6 in nasal lavage and LT-B4 in exhaled breath condensate) indicate subclinical effects of the exposure. Interestingly, the WS group had a 10-fold higher LT-B4 level in exhaled air before the exposure compared to the two other groups.

As the exposure may be characterized as moderate compared to real-life exposure at welding workshops, clinical effects at least in the two subgroups without a history of symptoms from the lower airways were not expected. Nor were heavier symptoms expected in the WS group as the inclusion criteria were symptoms from the lower airways at least once in the last month and as the group consisted of subjects without clear asthma—only one subject medicated more frequently for asthma-like symptoms (salbutamol inhalation).

The decrease in nasal symptoms during welding fume exposure was unexpected. To our knowledge this has not earlier been described. The reaction may be explained by a neurogenic mechanism as during treatment with camphor, eucalyptus or menthol (Burrow et al. 1983). Subjects at such exposure feel improved without effects on nasal resistance to airflow. In the present study, there was a tendency towards improvement in acoustic rhinometry mainly in the WS group. Thus, it may be hypothesized that such a reaction could play a role in development of lower airways symptoms as the exposure to the lower airways may increase.

In a previous study we found increased symptoms in welders during working days. The welders were in this study exposed not only to welding fumes, but also to particles from grinding (Jönsson et al. 2015). In the present study the participants were only exposed to welding fumes. This indicates that symptoms are either caused mainly by particles from grinding, or that the welding exposure in the chamber differed from that in the working places. The exposure was well reproduced during all experiments (Isaxon et al. 2013). The PM_2.5_ mean mass concentration was about 1 mg/m^3^ during the 5.5 h of exposure. As no particles above 2.5 µm were found, our results are comparable to the level of respirable particles as given in the Swedish occupational exposure limit (OEL) for inorganic dust (Swedish Work Environment Authority 2015). The exposure level is also in the range of that found in the Swedish welding industry (geometric mean: 1.3 mg/m^3^, minimum 0.1 mg/m^3^, maximum 38 mg/m^3^; Hedmer et al. 2014). In the present chamber study and in the earlier panel study in the workplaces, the ozone exposure was low; we therefore think that the effects come from the particles. However, as mentioned above in the welding industry dust exposure is also generated from other sources and thus containing particles with somewhat larger diameter than those in the welding fume.

The exposure level tested was chosen to secure a manganese (Mn) exposure below the OEL (respirable fraction 0.1 mg/m^3^, 8 h time-weighted, Swedish Work Environment Authority 2015).

Although iron was the dominant substance during exposure (70%), the level of Mn was just below the Swedish OEL (Isaxon et al. 2013). In a recent study of the Swedish welding industry, the exposure level of Mn was rather high (respirable fraction 0.08, <0.01–2.13 mg/m^3^, geometric mean, min–max) and more than 50% of the Mn measurements exceeded the occupational exposure limit (Hedmer et al. 2014). The critical effect of Mn is neurotoxic, but the effect on the airways is not as well investigated. Han et al. (2005) found that exposure to welding may cause changes in serum biomarkers of oxidative stress and an association between the serum level of manganese and manganese superoxide dismutase and glutathione peroxidase was found. Furthermore, Hałatek et al. (2005) saw that Club cell protein secretion was inhibited in younger welders not adapted to the manganese in shipyard welding. Thus manganese in welding fume may influence the airways.

Although no adverse clinical effects were registered in the present study, changes of several markers of inflammation were noticed during and after welding exposure, compared with filtered air. However, conclusions from such changes are not straight forward. Inflammation mediators may have a diurnal variation (Kendzia et al. 2012; Sennels et al. 2011). In this study, all exposure events followed the same time schedule and the results are calculated as the differences between exposure to welding fumes and filtered air. Thus, diurnal variations as explanations for the changes are not likely. Furthermore, some of the markers in the blood may be influenced by the hydration status of the participant which may differ between exposure to welding fumes and filtered air. However, as no changes were found in blood haemoglobin, this is not likely to explain the differences.

A limitation when many relationships are tested is the risk of false-positive findings (mass significances). We therefore focus our conclusions on findings where there are logical relationships between the changes in biomarkers and a reasonable biological hypothesis. However, the knowledge concerning the dynamics of inflammatory markers (especially when studied in different sampling media) is yet incomplete (Scarpa et al. 2014). As always, findings should thus be reproduced in other studies for confirmation.

Thus, we found an increase in blood neutrophils immediately after exposure to welding, most in the WS group. This may indicate an effect of the exposure. An increase in neutrophils was also found by Dewald et al. (2015) immediately after manual metal arc welding. The authors interpreted this as a sign of a subclinical inflammatory reaction.

The day after exposure the blood neutrophils decreased significantly, as did IL-8 in serum. IL-8 is a neutrophil attractant, thus an association may be expected. This is in contrast to the findings in firefighters after exposure (Greven et al. 2012), where an increase of both neutrophils and IL-8 in serum was noticed 24 h after firefighting. The reason for this discrepancy may be the kind or the intensity of exposure. Changes in inflammatory cytokines and leukocytes may occur within hours. Park et al. (1999) found that when subjects having a toluene diisocyanate associated asthma were challenged with TDI, serum neutrophil chemotactic activity significantly increased after 10 min of exposure and decreased after 60 min and then remained unchanged up to 7 h.

We found an increase in IL-6 in nasal lavage at the end of the exposure in both non-symptomatic groups. Similar results were shown in healthy volunteers 2 h after exposure to particles in various outdoor environments (Janssen et al. 2015). Furthermore, Stenfors et al. (2004) found a significant increase in IL-6 in bronchial lavage after exposure to diesel fume in healthy controls, but not in mild asthmatics. As IL-6 may play a role in the resolution process of inflammation (Xing et al. 1998; Fernando et al. 2014) these findings may indicate that non-symptomatic subjects may have a higher capability to stop the inflammatory process. A protective role of IL-6 has also been suggested in a study of firefighters from the World Trade Centre disaster (Cho et al. 2014) and is indicated also in some animal studies (Kopf et al. 1994; Wang et al. 2000; DiCosmo et al. 1994). The levels of IL-6 and IL-8 are in the same range as seen in an earlier study from our department (Xu et al. 2013), but the significant changes in the present study differ from changes found in the former study.

LT-B4 in EBC decreased during the welding exposure in the whole group. This mediator has chemo-attractant and furthermore activating properties to neutrophils and should thus develop parallel to IL-8. IL-8 decreased later, but it was measured in serum. Our finding is in contrast to that in ozone-sensitive subjects who increased in LT-B4 after ozone exposure (Alfaro et al. 2007). The most remarkable result was that the symptomatic welders before exposure had a tenfold higher pre-exposure level of LT-B4 at both exposure events than the other two groups. An effect on LT-B4 in EBC in welders has earlier been suggested (Hoffmeyer et al. 2012; Mittmann-Frank et al. 2011), but to our knowledge, elevated levels have not been associated with symptoms in the welders previously.

Taken together our interpretation of the results is that the rapid decrease in LT-B4 in EBC is a true effect of exposure to welding fume nanoparticle aggregates, followed by a subsequent decrease in neutrophils and IL-8 in blood. In a sensitive subgroup of welders with work-related airway symptoms, however, chronic exposure to welding fumes may lead to increased EBC LT-B4 levels, reflecting an ongoing neutrophilic low-grade inflammation. This sensitive group is also characterized by a tendency towards nasal opening during welding exposure, which could be a reason for increased sensitivity to welding fumes. Another difference between the sensitive group and the asymptomatic groups is the increase in IL-6 after acute exposure in the latter groups, suggesting that the IL-6-response is part of a healthy response that leads to the resolution of the local inflammation after exposure. While the causal chain is still not clear, this study thus finds that those who have developed symptoms in the lower airways from long-term exposure to welding fumes differ from the other participants in their expression of inflammatory cytokines. These differences in immune responses should be studied further to determine the mechanisms through which welding fumes can affect health, as should the possibility of using of LT-B4 in exhaled air as an early biomarker of airway effects in welders.

In conclusion, although no adverse clinical effects could be shown at welding fume exposure well below the Swedish exposure limit (OEL 5 mg/m^3^ 8 h TWA respirable dust) and other European exposure limits, responses in the inflammatory system may indicate a risk for adverse effects in the airways at such exposure.
